# Evidence of nanoemulsion as an effective control measure for fruit flies *Drosophila melanogaster*

**DOI:** 10.1038/s41598-019-47045-3

**Published:** 2019-07-22

**Authors:** Sudhakar Krittika, P. Indhumathi, B. N. Vedha Hari, D. Ramya Devi, Pankaj Yadav

**Affiliations:** 1 Fly Laboratory # 210, Anusandhan Kendra-II, School of Chemical & Biotechnology, SASTRA Deemed to be University, Thanjavur, 613401 Tamil Nadu India; 2 Pharmaceutical Technology Laboratory # 214, Anusandhan Kendra-II, School of Chemical & Biotechnology, SASTRA Deemed to be University, Thanjavur, 613401 Tamil Nadu India

**Keywords:** Behavioural ecology, Entomology

## Abstract

Pesticide resistance is a common concern. It exerts close association with economic and health associated problems in various plants and other organisms. Several approaches have been trialled for attracting and trapping the insects and flies that are acting as vectors for transmission of communicable diseases. Although *Drosophila melanogaster* (fruit flies) is not an agricultural pest, its presence in consumer dwelling areas is an objection to human, as it indicates signs of an unhealthy environment or products. The current study focuses on the development of nanoemulsion with synthetic attractants and entrapping in sticky glue formulation that could provide prolonged effect for attracting and trapping the fruit flies. The results of our study showed the efficient attractive ability of exposed nanoemulsion (A3E1T) containing amyl acetate, ammonia, ethanol and Tween 80 compared to that of control. While the sex-based effect was not very prominent, the nanoemulsion showed a higher relative response index to the flies and increased activity even during their siesta time. Therefore, the nanoemulsion-based approach could be identified as one of the promising lines of attack and a suitable alternative for the existing fruit fly control measures. The present study is the first of its kind in reporting the ability of nanoemulsion formulation to attract and influence the activity of fruit flies *D. melanogaster*, up to our best of knowledge.

## Introduction

Pests are living organisms belonging to the phylum Arthropoda, known to cause significant economic losses in agriculture, food industry and prove as a vector for transmitting disease-causing pathogens to other organisms, especially human beings^1–3^. Since a long time, people are being affected by the interference of a variety of mites and insects. Application of leaves obtained from certain Malvaceae plants was found to be effective against mites, and thereby led to the concept of repellent and attractant usage^4,5^. Since then, the search for efficient repellents and attractants for pests, insects, and mites have gained momentum.

*Drosophila melanogaster*, commonly named as fruit flies (belonging to the family Drosophilidae) has an average lifespan of 50–60 days. *D. melanogaster* is not an agricultural pest^6^, unlike the other fruit flies from closely related family Tephriditae that are capable of mounting extensive crop damage. However, their presence in showrooms, markets, and food serving areas is an objection by the consumers, as they are thought to suggest an unhealthy environment or products. The spotted-wing fly, *D. suzukii* (a closely related species of *D. melanogaster*) infects soft-skinned fruits like berries and peaches by piercing the skin of the matured fruits and laying eggs on them, which make the fruits unfit for consumption^7,8^. This could lead to an indirect gradual infestation by other *Drosophila* species and also promote microbial decay of the fruits.

To combat the intrusion of these pests, insecticides are used in the markets and other consumer dwelling areas as a control measure; nevertheless, many countries impose severe regulations in the use of insecticides. These pesticides remain as residual deposits on foods or animal feeds and are vulnerable to the environment^9^. Apart from insecticides, there are various commercially available attractants that can be used effectively without harming other animals or humans and so, employed as an environmentally safe measure. However, the commercially available attractants have limitations due to moderate effectiveness, high cost and lesser effect with respect to time. Hence, the development of an attractant to serve as an efficient trap with the prolonged effect is highly essential^4^. This lacuna could be bridged by the use of nanotechnology, based on the formulation development of various nanoemulsions and nano pesticides using both synthetic and biological materials. The present study focuses on formulation of the nanoemulsion with synthetic attractants that could provide prolonged effect through attraction of the flies.

Emulsions are formed when two immiscible liquids are dispersed into one another^10^ in the presence of an emulsifying agent. Based on the size of globules formed during emulsification and its thermodynamic stability, it can be categorized as conventional emulsion and nanoemulsion^11–13^. The conventional emulsions are creamy white, while the nanoemulsions are translucent due to their smaller globular size ranging between 1 nm to 100 nm, which confers higher kinetic stability and solubilising capacity. Researchers have reported nanoemulsions with the globule size up to 500 nm with moderate stability for suitable applications^12,14^. They play a significant role in the bioengineering field due to specific characteristics that are utilized for the protection of substances, prolonged effect, controlled release, targeting approach, etc.^14,15^. Further, when surfactants are used in insecticide formulations, they can also affect insecticidal activity apart from aiding in emulsion formation^16^.

The model organism (*D. melanogaster*) used for this study is known to have an unique olfactory mechanism to recognize food source and nutrient-rich sites^17^, while its larvae have shown increased attraction for short chain acetates and repulsion for long-chain acetates^18^. Apart from the synthesis of nanoemulsion of the attractants, a trapper is also required to prevent the escape of insects and to control monitor their presence. Hence, the use of resin like colophony that serves as a sticky substrate could provide better efficiency when the synthesized nanoemulsion of the attractants is loaded in it.

In the present study, we aim to develop a nanoemulsion with attractants and load in the colophony resin trap, which will not produce any harsh effects to the environment, while its efficacy to attract other insects can also be tested. The formulations were trailed with and without the addition of emulsifying agents (Span 80 and Tween 80) to obtain the nanoemulsion and homogenous mixtures, respectively. The emulsifying agents were selected based on the stability to obtain water in oil (w/o) or oil in water (o/w) system. The composition of the formulations include amyl acetate as a major component because of its inherent fruity odour, while the other components were selected based on previous reports of researchers, wherein synthetic chemicals were used to attract other insects like mosquitoes^19–21^. The prepared mixtures and nanoemulsions were tested for their ability to attract *D. melanogaster* at their two different phases of activity/rest rhythm (i.e., morning and evening). The changes in their activity patterns due to the sample exposure were monitored and plotted as actograms, which showed increased activity upon the sample exposure. The results of our study showed that the fruit flies were attracted to the test samples and their response index was varying with respect to the sample type and time of the day (morning or evening), while the effect of different sex was not very prominent. Also, the activity of flies increased during its exposure to the sample, which confirmed their response to the attractant chemicals used in the study. Thus, these studies could be considered as preliminary evidence to utilize nanoemulsion formulation to attract and trap fruit flies in commercial markets and the local environment. Based on the literature survey, we could evidence that this approach is reported for the first time as we have clearly demonstrated the ability of the nanoemulsion system to attract the fruit flies, *D. melanogaster*.

## Results

### Characterization studies

Prior to the live experiments with the fruit flies, preliminary physicochemical characterization studies such as size, stability, pH, density and viscosity of the prepared nanoemulsion were performed (Table 1).

**Table 1:** Assessment of physical parameters of test nanoemulsions.

Nanoemulsion Formulation Trial	Average globule Size (nm)	PDI	Zeta Potential (mV)	pH	Density (g/mL)	Viscosity (cP) @ 27 °C
A3E1S	165	0.257	−31.6	10.20	0.892	3.03
A3E1T	65	0.147	−28.1	10.75	0.895	3.605
A3E2T (o/w)	359	0.410	−30.2	10.66	0.922	4.03

The nanoemulsions formed were tested for pH, density and viscosity.

The formulated nanoemulsions exhibited globule size from 60–360 nm and polydispersity ranging from 0.1 to 0.4. The zeta potential value within the narrow range of −28 to −32 indicated greater stability of the preparations. The pH of all the samples was found to be alkaline (around pH 10.2 to 10.6) due to the presence of amine component as major ingredients. The density ranging within 0.8–0.9 g/mL and viscosity as 3.0–4.0 cP at ambient room temperature, which confirmed its ideal adhesiveness and restricted flow of the formulations suitable for further attraction studies. Among the three formulations, the sample A3E1T could be considered as optimum preparation, which could be attributed to its globule size of 65 nm, PDI of 0.147 (highly monodispersed) and zeta potential of −28.1 mV with ideal pH, density and viscosity.

### Chemotactic response

**Figure 1:**
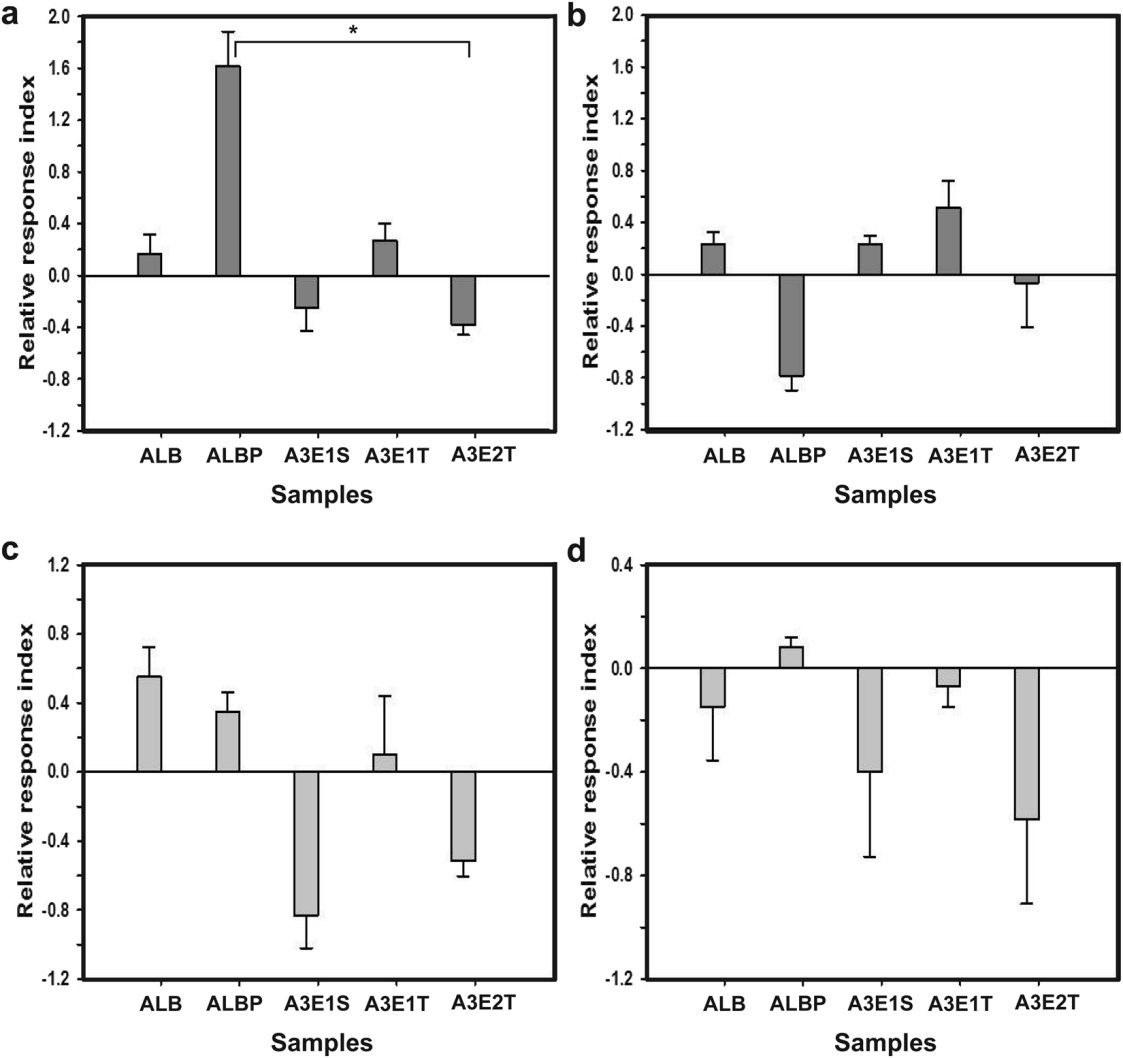
Chemotactic assay. The chemotactic behaviour of flies upon exposure to different test samples in the morning (males, **a**; females, **b**) and evening (males, **c**; females, **d**) are indicated as their Relative Response Index (RRI). The error bars are standard error around the mean (SEM) and the statistically significant differences are indicated by asterisks. The statistical analysis was done by Kruskal-Wallis ANOVA followed by multiple comparisons of mean ranks of all groups.

The efficacy of five different samples towards fruit flies (2 mixtures and 3 nanoemulsions) was tested at ZT2 (Zeitgeber Time; ZT henceforth) and ZT10 using counter current apparatus. Kruskal-Wallis ANOVA on the relative response of fruit flies showed significant effect of samples in males [morning (H_*4,15*_ = 11.27, p = 0.02)], while the others [males- evening (H_*4,15*_) = 9.36, p = 0.05); females (morning (H_*4,15*_ = 6.37, p = 0.17; evening (H_*4,15*_ = 2.96, p = 0.56) did not show significant effect of the samples. Multiple comparisons of mean ranks of all groups’ show significant effect of samples only in the morning in males (Fig. 1a–d). The Kruskal-Wallis test also revealed that there is no significant effect of the samples in the morning when both the males and females were taken together for their response (Supplementary Fig. 1). In the morning (males), the samples of ALBP (amyl acetate, lactic acid, butanone, propionic acid) and A3E2T (o/w; amyl acetate, ammonia, butanone, ethanol, tween 80, water) alone are significantly different (Fig. 1a). Thus, the results of the chemotaxis assay suggested that flies responded to the test samples irrespective of sample preference in the morning and evening, while the males alone showed considerable difference in their preference to the samples.

### Attraction study

**Figure 2:**
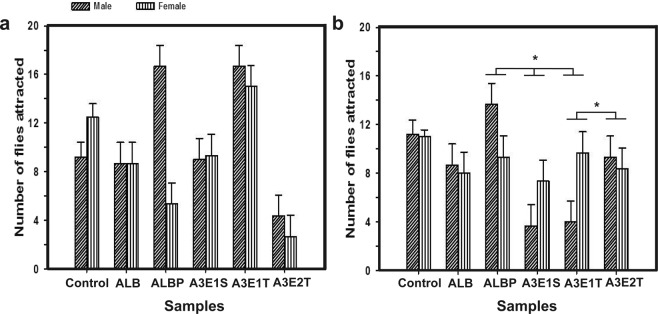
Attraction study. The number of flies attracted towards different test samples in the morning (**a**) and evening (**b**) are indicated, while the total number of flies was 20. The error bars are standard error around the mean (SEM) and the statistically significant differences are indicated by asterisks. The statistical significance is reported based on two-way ANOVA followed by post hoc multiple comparisons by Tukey’s HSD test.

Attraction study was assessed by the measure of the number of flies attracted towards the test samples within a specific exposure time (10 minutes). ANOVA performed on the number of flies attracted towards each of the subjected samples showed no significant effect of the sample (N; F_*5,29*_ = 2.49, *p* < 0.053; Fig. 2a) and sex (S; F_*1,29*_ = 0.002, *p* < 0.967; Table 2) in the morning. However in the evening, the flies’ response was observed to be significantly different towards the samples (N; F_*5,29*_ = 6.81, *p* < 0.0003), even though, the effect of sex was not significant (F_*1,29*_ = 0.02, *p* < 0.883; Table 2). During the evening, the attraction towards various samples was significantly different (Fig. 2b), wherein the flies were attracted towards ALBP, A3E1S (amyl acetate, ammonia, butanone, ethanol, span 80, water), A3E1T (amyl acetate, ammonia, butanone, ethanol, tween 80, water) and A3E2T (o/w). Post hoc multiple comparisons using Tukey’s HSD test revealed that the number of flies attracted at different time of the day (T; F_*1,48*_ = 12.98, *p* = 0.0007; Fig. 2a,b), towards samples (F_*5,48*_ = 19.99, *p* < 0.0001), T × N (F_*5,48*_ = 2.73, *p* = 0.0298), N × S (F_*5,48*_ = 22.19, *p* < 0.0001) and T × N × S (F_*5,48*_ = 5.47, *p* = 0.0005) were all significantly different, while the effect of sex (F_*1,48*_ = 0.039, *p* = 0.84) was not significant. Further, in order to check whether the differential response of flies was due to the time of the day, rather than the sample itself, we analysed the response of flies in the control. The flies attracted in the control was not significantly different at the two time-points of the day (F_*1,9*_ = 1.4, *p* = 0.27), while the sex-based effect (F_*1,9*_ = 12.59, *p* < 0.006) was significant, thereby stating that response of flies to the samples is time-based as well. Statistical analysis showed that the number of flies attracted to various samples and the interaction among sample and sex was significantly different, while the effect of sex was significantly not different (Fig. 2b). Thus, the results of attraction assay suggested that the response of the flies was variable according to the time of the day for the exposure of same test sample.

**Table 2:** ANOVA and post-hoc multiple comparison details of the different assays performed under LD12:12 hr.

Assay	Effect	d.f.	MS effect	d.f. error	MS error	*F*	*P*<
Attraction study	Time of the day (T)	1	42.014	48	3.236	12.983	0.0007
	Sample (N)	5	64.692	48	3.236	19.991	0.0001
	Sex (S)	1	0.125	48	3.236	0.039	0.845
	T × N	5	8.847	48	3.236	2.734	0.0298
	T × S	1	0.014	48	3.236	0.004	0.948
	N × S	5	71.825	48	3.236	22.195	0.0001
	T × N × S	5	17.714	48	3.236	5.474	0.0005
Time taken for complete inactivity	Time of the day (T)	1	109.35	40	3.7	29.554	0.0001
	Sample (N)	4	4.69	40	3.7	1.268	0.2986
	Sex (S)	1	58.02	40	3.7	15.68	0.0003
	T × N	4	7.22	40	3.7	1.953	0.1205
	T × S	1	0.82	40	3.7	0.221	0.641
	N × S	4	463.14	40	3.7	125.173	0.0001
	T × N × S	4	14.94	40	3.7	4.038	0.0076

### Time taken for complete inactivity

**Figure 3:**
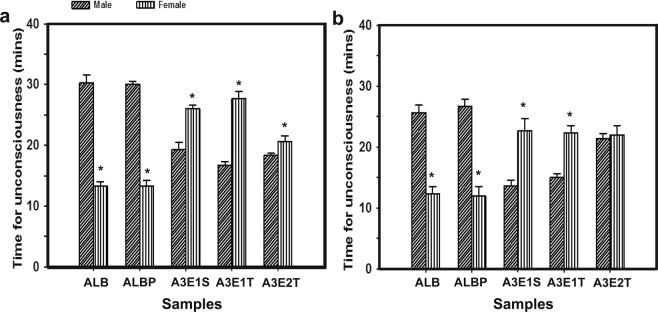
Time taken for complete inactivity. The time taken (in minutes) for all the flies in each set of the test sample, to get completely inactive in the morning (**a**) and evening (**b**) are indicated. Other details are as same in Fig. 2.

Although the above experiments showed attraction of the flies to the samples within the specified exposure time of 10 minutes, we focused to check the response of flies for a longer exposure (12–30 minutes) to the test samples. In this experiment, the time taken for the flies in each trial to become completely inactive was noted. The loss of consciousness of the flies was an indication of the effectiveness of the tested samples for prolonged exposure time and higher concentration. The time taken for the flies to exhibit inactivity in response to the exposed sample showed a trend of insignificant differences with respect to samples (morning- F_*4,24*_ = 0.19, *p* < 0.94; evening- F_*4,24*_ = 0.31, *p* = 0.87) and sex (morning- F_*1,24*_ = 1.23, *p* < 0.28; evening- F_*1,24*_ = 1.02, *p* < 0.32; Fig. 3a,b). Post hoc multiple comparisons using Tukey’s HSD test revealed that the number of flies attracted in accordance with the time of the day (F_*1,40*_ = 29.55, *p* < 0.0001), sex (F_*1,40*_ = 15.68, *p* = 0.0003), N × S (F_*4,40*_ = 125.17, *p* < 0.0001) and T × N × S (F_*4,40*_ = 4.04, *p* = 0.008; Table 2) were significantly different, while the effect of sample (F_*4,40*_ = 1.27, *p* = 0.29) was not significantly different. Therefore, the results of this study suggested that prolonged exposure of these test samples could result in an inactive state of the flies, which confirmed the formulated nanosuspension that could be used effectively to attract and anesthetize the flies.

### Activity changes by mixture and nanoemulsion exposure

**Figure 4:**
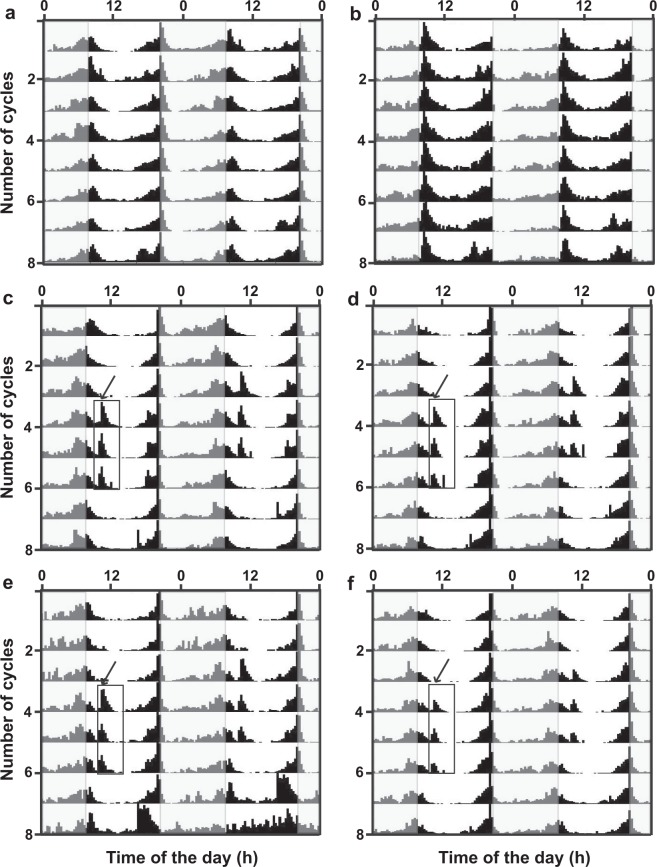
Response of flies upon exposure to mixtures. The double-plotted actograms shows activity of male (**a,c,e**) and female (**b,d,f**) flies in control and mixtures- ALB and ALPB respectively. The flies respond by increasing their activity upon exposure to the mixtures at ZT2 and ZT10 (where the lights on at 08:00 hr & lights off at 20:00 hr). The x-axis represents time of the day and y-axis represents the number of cycles. The white and gray shaded regions represent light and dark phases of LD (12:12) regime of the assay respectively. The enclosed three cycles activity peaks in the box shows the increased activity upon nanoemulsion, while the arrow indicates the time of the sample exposure.

**Figure 5:**
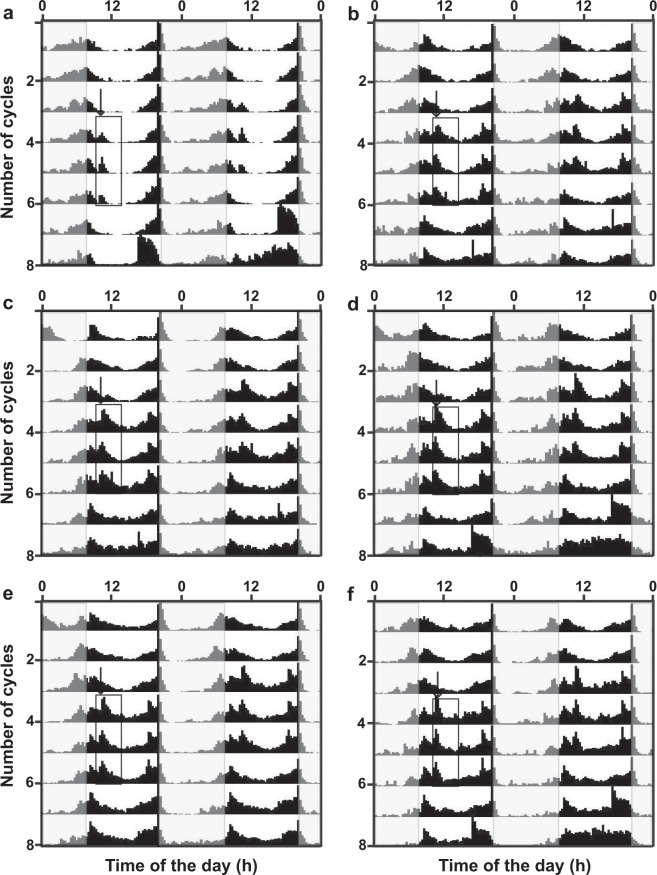
Response of flies upon exposure to nanoemulsions. The double-plotted actograms shows activity of male (**a,c,e**) and female (**b,d,f**) flies to the nanoemulsions of A3E1S, A3E1T and A3E2T respectively. The flies respond by increasing their activity upon exposure to the nanoemulsions and disturbed siesta time, while the control for the experiment and other details are same as that of Fig. 4.

The represented double-plotted actograms of virgin males and females showed the increase in activity as a response for exposure to different mixtures and nanoemulsions (Figs 4c–f, 5a–f; respectively). The actograms for the mixtures ALB (Fig. 4c-males, 4d-females) and ALBP (Fig 4e-males, 4f-females) confirmed the increase in flies response and activity immediately after the exposure at ZT2 and ZT10. Additionally, their siesta time between ZT4-ZT6 was not disturbed, wherein they have recorded the lowest activity at that time similar to before sample exposure and control (Fig. 4a,b) Also, the males and females respond equivalently to the mixtures (Fig. 4c–f). Upon encountering with nanoemulsion A3E1S (Fig. 5a,b), A3E1T (Fig. 5c,d) and A3E2T (Fig. 5e,f), the activity of flies increased immediately similar to the mixtures. However, the striking difference appeared in fact, when these flies had comparatively higher activity in their siesta time as well. This disturbance was prominent upon exposure to A3E1T and A3E2T (Fig. 5c–f). The nanoemulsion exposure had not induced any arrhythmicity in the activity of the flies (Fig. 5), and therefore showed intact normal functioning circadian clock. Thus, these results clearly demonstrated the response of the flies exposed to the formulated samples through the enhancement of their activity levels.

## Discussion

The presence of *D. melanogaster* in various consumer dwelling areas leads to several objections raised by the human population, and the use of pesticides or insecticides is not a healthy option as the continuous use of these substances renders resistance in insects apart from contaminating the environment^9,22^. Nanoemulsions are identified as one of the alternative approaches, because of their stability as compared to emulsions, steady release, prolonged effect and relatively easy method of synthesis^14,15,23^. Hence, the present study focused on the formulation of nanoemulsion with synthetic attractive chemicals that could provide the prolonged effect as the fruit flies attractant. The mixtures and nanoemulsions used in our study consisted of a blend of different compounds, wherein amyl acetate was constant, due to its fruity odour and also by the suggestions provided by certain studies that explained the attraction effect of amyl acetate based on its intensity^24,25^.

The olfactory response of *D. melanogaster*^24,26^ has the capability to influence several processes including feeding, life-history traits^27^, social behaviour^28^, etc. Hence, studying their olfactory response to various attractants was employed as a suitable method of analysis. The significant difference in relative response index (RRI) of flies upon exposure to mixtures and nanoemulsions (varying in a single chemical compound) was observed in males only in the morning (Fig. 1a–d).

Further, the number of flies attracted towards the samples was comparatively high in the mixture ALBP (Fig. 2b) than others, while the time taken for them to become fully inactive was not different (Fig. 3a,b). The attraction to ALBP was higher due to the presence of propionic acid (as it is a common odour in *Drosophila* food substrates) as these flies were already known to be attracted towards propionic acid^29^. Among the nanoemulsion formulations, A3E2T (o/w) sample exhibited higher attraction in both the sex during the evening and was relatively significant. However, these results were not consistent in the morning, as this difference for an olfactory response might be due to the phase difference in morning activity bout governed by the endogenous circadian clock^30^ and also by the way the flies responded to the same samples at a different time of the day. Our results were comparable to a previous report^30^, which stated that circadian rhythms were necessary to regulate olfactory response that influenced the social behaviour as well. The reduced differential morning response towards the samples was similar to the report suggested by Zhou *et al*.^31^. This study had begun as an initial step towards the identification of potential attractants for the pests.

The actograms of the male and female *D. melanogaster* displayed the enhanced activity during the exposure to nanoemulsion, as an integrated olfactory response towards the samples due to their fruity smell (^32^; Fig. 5a–f). The response in the flies’ activity has been highly influenced by the nanoemulsion formulations due to their stronger odour compared to the mixture preparations (Fig. 4c–f). The flies exposed to nanoemulsions showed enhanced activity not only during the activity phase but also in their siesta time (Fig. 5a–f). The actograms also showed that the flies have not entrained to these exposures possibly because three cycles of nanoemulsion exposure might not be sufficient for the same. Moreover, such a response to the change in the behaviour of the flies based on the concentrations of odorants was found to be dose-dependent^33^. Further, challenges of these results outlooks towards the need of in-depth understanding of the role of circadian clock system neurons especially for the nanoemulsion exposure, expression level of pigment dispersing factor (PDF- a neuropeptide) expressed by clusters of ventrolateral neurons (LNv) the principal circadian pacemakers^34^, even though olfactory response does not require PDF^31^.

In terms of commercialization of these mixtures and nanoemulsions, we expect to observe no sex-based effect. Hence, our formulations would be effective against both male and female as a whole, rather than focusing more towards attracting the females as they are capable of laying eggs and spoiling the fruits. Therefore, we comprehend the optimized nanoemulsion formulation for the specific application, as the results confirmed the importance of each component (amyl acetate, propionic acid, and ammonia) for the metabolic and physiological benefits of both male and female flies. The nanoemulsion can be loaded on colophony resins and can be used as a sticky glue system that can be effective in trapping the flies. Consequently, these confer an advantage of unbiased attraction towards male and female flies and the future outcome of this study would be well-established, once the commercial approval and the optimized composition are patented.

## Methods

### Flies rearing

Fruit flies *D. melanogaster* was reared on corn, yeast agar medium at 25 ± 1 °C on 12:12 h light:dark cycles (light- 8:00 to 20:00 h). Three days old flies were used for all the experiments. Freshly emerged flies were separated as male and female by short CO_2_ anaesthetization, in order to avoid interference of CO_2_ while conducting the behavioral assays.

### Chemicals used

Lactic acid, Propionic acid, Amyl acetate, Toluene, and Butanone were purchased from Merck Specialities Pvt. Ltd. and Loba Chemie Pvt. Ltd. and used as the attractive chemicals in this study. The emulsifying agents, Span 80 and Tween 80; and the Co-surfactants namely Octan-1-ol and Ethanol were procured from SD fine Chem. Ltd. Colophony resin was procured from a local market.

### Preparation of nanoemulsion and mixtures

The number of ingredients used for the preparation of nanoemulsion and the simple mixture were trialled experimentally and the composition of the selected formulations are shown in Table 3. The primary emulsion was prepared by the drop-wise addition of non-aqueous phase (Toluene / Amyl acetate) to aqueous phase (Lactic acid/ Propionic acid/ Ammonia/ Butanone) in presence of the emulsifying agent (Span 80/ Tween 80), placed under ultra-probe sonicator (Vibronics, Mumbai) at 100 V for 5–10 minutes. The two immiscible liquids formed a homogenous milky white dispersion, which confirmed the formation of emulsion. Since, the non-aqueous solvent toluene demonstrated high instability issues to form an emulsion, the formulations prepared using amyl acetate alone were considered for further experiments. The prepared primary emulsion was placed in magnetic stirrer maintained at 500 rpm and co-surfactant (ethanol/ octan-1-ol) was added drop-wise using a syringe within 10–15 min. The milky white dispersion converted into a transparent emulsion, due to the size reduction of the macro-sized globules of the primary emulsion to nano-sized globules in the yielded nanoemulsion (A3E1S, A3E1T, and A3E2T). In the case of the mixture preparations (ALB and ALBP), the required ingredients were added by uniform geometric mixing and stirred in magnetic stirrer at 200 rpm for 5 minutes. The formulated nanoemulsion was characterized for size, polydispersity index (PDI), zeta potential, pH, density and viscosity to ensure their physical stability and suitability for the study^35^.

**Table 3:** Composition of the formulations.

Formulation Type	Miscible Mixtures		Nanoemulsions		
Materials used (mL)	ALB	ALBP	A3E1S (w/o)	A3E1T (w/o)	A3E2T (o/w)
Amyl acetate	1.5	1	1.5	1.5	1.5
Lactic acid	1.5	1	—	—	—
Butanone	2	1	1.5	1.5	1.5
Propionic acid	—	1	—	—	—
Ammonia	—	—	2	2	2
Span 80	—	—	0.2	—	—
Tween 80	—	—	—	0.2	0.2
Ethanol	—	—	2.5	2.5	4
Water			0.5	0.5	11
Total volume of the formulation (mL)	5	4	8.2	8.2	20.2

The table shows the designated name, composition and nature of the test samples used for the studies.

### Particle size and zeta potential

The particle size and zeta potential of the nanoemulsion was measured by electroacoustic dynamic light scattering principle using Zetasizer (Malvern Nano series ZS, UK). The samples were analyzed within 24 hours of preparation (triplicate).

### pH

The pH of the formulated nanoemulsion was measured using a pH meter, calibrated using buffer solutions of pH 4.0 and 9.1. The electrode of the pH meter was immersed in the sample and the pH was noted down.

### Density

The density of each nanoemulsion was estimated by weighing accurately 5 mL of the sample in a standard pycnometer using an electronic weighing balance. The empty weight of pycnometer was subtracted from the obtained weight of pycnometer with the sample to obtain an actual mass of 5 mL of the nanoemulsion. The obtained mass per unit volume indicated the density value.

$${\rm{Density}}=\frac{{\rm{Mass}}}{{\rm{Volume}}}$$

### Viscosity

Viscosity for the prepared nanoemulsion was measured in Brookfield Viscometer with the spindle no. 63 and at varying rpm such as 200, 150, 100, 60, 50, 30 and 20. Since viscosity could be measured as a function of temperature, the experiment was performed at ambient room temperature 27 °C for all the samples.

### Chemotaxis assay

Attraction studies were conducted by separating the flies based on their sex, while each sample was tested with a set of 20 flies each. Since the direct exposure of prepared mixtures and nanoemulsions caused complete inactivity in the flies within less than 5 minutes of exposure, the formulations were diluted to form an effective 30% solution (30 μl of sample + 70 μl of water) which was further used for all the experiments. For the volume of the vial of 28.27 cm^3^, 100 μl of the sample of the prepared 30% sample was used. The assay were performed using counter current apparatus in a closed room with light intensity of ~150 lux at room temperature ~25 °C. Also, *D. melanogaster* has the capacity of detecting the odour of attractants at low concentrations^36^. For each sample and each time of the day, the experiment was carried out with a different set of flies, in order to avoid possible adaptation in the flies due to prolonged or repeated exposure to the test samples^37^.

Chemotaxis study was performed using counter current apparatus (Supplementary Fig. 2a,b) designed by Seymour Benzer^38^, wherein the whole experiment comprised three trials and each trial for each nanoemulsion involved 5 chances. In every chance, flies were exposed with attractants for 15 seconds and allowed to move towards it, wherein the flies could move from vial V_0_ to V_1_ in the first chance. Once the 15 seconds of exposure was completed the shutter of counter current apparatus was closed for the next 15 seconds, whereby flies present in V_1_ had reached zero distance in that chamber and the remaining flies (out of total 20) were available in V_0_ itself. During the second chance, the shutter of counter current apparatus was opened for 15 seconds and flies were allowed to move towards the attractant (from vial V_1_ to V_2_) in a similar manner. At the same time, the remaining flies present in V_0_ could move to V_1_. The same protocol was repeated for 3^rd^, 4^th^, and 5^th^ chances so that the flies moved from V_2_ to V_3_, V_3_ to V_4_ and V_4_ to V_5_, respectively. The whole experiment was repeated for three trials and the response of flies for each sample was evaluated to select the optimum nanoemulsion, for which the flies showed a high response. The response index of flies on exposure to the samples (experiment) and in their absence (control) were assayed, to calculate their relative response index (RRI) in both male and female during morning and evening at ZT2 (2 h after lights-on) and ZT10 (2 h before lights-off). A positive RRI indicated attraction of the flies towards the sample, whereas a negative RRI indicated repulsion for the same.

$$\begin{array}{l}{\boldsymbol{ResponseIndex}}\,({\boldsymbol{RI}})=\frac{0\times {V}_{0}+1\times {V}_{1}+2\times {V}_{2}+3\times {V}_{3}+4\times {V}_{4}+5\times {V}_{5}}{{V}_{1}+{V}_{2}+{V}_{3}+{V}_{4}+{V}_{5}}\\ {\boldsymbol{RelativeResponseIndex}}({\boldsymbol{RRI}})=R{I}_{experiment}-R{I}_{control}\end{array}$$

### Attraction assay and time taken for complete inactivity

Individual experiments were designed to assess the effect of each nanoemulsion by evaluating the number of flies attracted and the time taken for all the flies to become inactive. For the attraction assay, the flies were exposed to each of the test samples for a limited time of 10 minutes. The time taken by all the flies involved in a batch study to become completely inactive during the sample exposure was monitored visually and noted as the time of complete inactivity. For both these assays, a total of 20 flies (20 male and 20 female tested separately for the effect of sex) were taken in a batch for each sample at both ZT2 and ZT10 (2 times of the day × 5 samples × 2 sex × 3 trials).

### Activity assay

The activity assay of the flies was performed using *Drosophila* activity monitors (DAM) from Trikinetics, MA, USA. The DAM system is a computer-aided device that uses infrared emitter and sensor to detect the movement of individual flies in a narrow glass tube (Trikinetics, USA). Randomly, 32 virgin male and female flies from the population were loaded separately into glass tubes (5 mm diameter × 7 cm length) with *ad libitum* food. Their locomotor activity was monitored for 8 days under LD (Light:Dark 12:12 h). The flies were exposed to the nanoemulsion and mixture samples for 10 minutes in separate boxes and observed during the morning and evening time (ZT2 and ZT10) to monitor the effect of the time of the day on the activity of fruit flies. The limited 10 minutes exposure was given to observe the changes in the flies’ activity upon exposure to samples and to check whether the samples induced inactivity or hyperactivity in flies. The readings of physical conditions and flies inactivity/hyperactivity recordings were saved regularly in every 5 minutes bin. The activity data of the flies were analysed and plotted (actograms) by CLOCKLAB (Actimetrics, USA). Physical conditions such as temperature (~25 °C) and relative humidity (~70%) inside the recording metallic box (15 × 10 × 8 cm^3^; provided with LED lights) placed inside a cubicle were monitored every 5 minutes using *Drosophila* Environmental Monitor (DEnM) and were found to be stable throughout the assay. The volume of sample used for this assay was 4.2 ml (of each of the tested sample of the same dilution), so as to maintain the exposure range according to the volume of the box (10 cm × 15 cm × 8 cm).

### Statistical analysis

The results were expressed as the mean ± SEM, wherever appropriate. All statistical analysis was performed by Kruskal-Wallis ANOVA (chemotaxis assay) and two-way ANOVA (attraction study and time taken for complete inactivity assay) in STATISTICA using Windows Release 7 (StatSoft Inc. 1995, 2004). The fixed factors such as samples (N), sex (S) and time of the day (T) were all considered simultaneously for two-way ANOVA, while for Kruskal-Wallis ANOVA these fixed factors were considered individually. Further, the post hoc multiple comparisons of Tukey’s HSD done were done for two-way ANOVA, while multiple comparisons of mean ranks of all groups were done for Kruskal-Wallis analysis.

## Supplementary information

Supplementary figures

## References

[1] Franco JR, Simarro PP, Diarra A, Jannin JG (2014). Epidemiology of human African trypanosomiasis. Clin. Epidemiol..

[2] Scott JG (2014). Genome of the house fly, *Musca domestica* L., a global vector of diseases with adaptations to a septic environment. Genome Biol..

[3] Cholewiński M, Derda M, Hadaś E (2017). Hygiene pests as vectors for parasitic and bacterial diseases in humans. Ann. Parasitol..

[4] Dethier, V. G. Introduction in *Chemical Insect Attractants and Repellents*. 1–11 (Lewis, H. K. & Co. Ltd., 1947).

[5] Debboun M, Strickman D (2013). Insect repellents and associated personal protection for a reduction in human disease. Med. Vet. Entomol..

[6] Emameh RZ, Syrjänen L, Barker H, Supuran CT, Parkkila S (2015). *Drosophila melanogaster*: a model organism for controlling Dipteran vectors and pests. J. Enzyme Inhib. Med. Chem..

[7] Lee JC (2011). The susceptibility of small fruits and cherries to the spotted-wing drosophila, *Drosophila suzukii*. Pest Manag. Sci..

[8] Rota-Stabelli O, Blaxter M, Anfora G (2013). *Drosophila suzukii*. Curr. Biol..

[9] Simon-Delso N, Amaral-Rogers V, Belzunces LP, Bonmatin JM (2015). Systemic insecticides (neonicotinoids and fipronil): trends, uses, mode of action and metabolites. Environ. Sci. Pollut. Res. Int..

[10] Khan BA (2011). Basics of pharmaceutical emulsions: A review. Afr. J. Pharm. Pharmacol..

[11] Solans, C. *et al*. Nanoemulsions: formation and properties in *Surfactants in solution: fundamentals and applications, Surfactant Science* Series (ed. Shah, D., Moudgil, B. & Mittal, K. L.) 525–554 (Marcel Dekker, 2002).

[12] Tadros T, Izquierdo P, Esquena J, Solans C (2004). Formation and stability of nano-emulsions. Adv. Colloid Interface Sci..

[13] Komaiko JS, Mc Clements DJ (2016). Formation of food grade nanoemulsions using low-energy preparation methods: A review of available methods. Compr. Rev. Food Sci. Food Saf..

[14] Zeeb B, Herz E, Mc Clements DJ, Weiss J (2014). Impact of alcohols on the formation and stability of protein-stabilized nanoemulsions. J. Colloid Interface Sci..

[15] Hörmanna K, Zimmer A (2016). Drug delivery and drug targeting with parenteral lipid nanoemulsions-A review. J. Controlled Release..

[16] Singh A, Van Hamme JD, Ward WP (2007). Surfactants in microbiology and biotechnology: Part 2. application aspects. Biotechnol. Adv..

[17] Gerber, B., Biernacki, R. & Thum, J. Odor-taste learning assays in *Drosophila* larvae. Cold Spring Harb Protoc. **2013** (2013). 10.1101/pdb.prot071639 23457337

[18] Cobb M, Dannet F (1994). Multiple genetic control of acetate-induced olfactory responses in *Drosophila melanogaster* larvae. Heredity (Edinb)..

[19] Qiu YT (2007). Attractiveness of MM-X traps baited with human or synthetic odor to mosquitoes (Diptera: Culicidae) in The Gambia. J. Med. Entomol..

[20] Verhulst NO (2011). Improvement of a synthetic lure for *Anopheles gambiae* using compounds produced by human skin microbiota. Malar. J..

[21] Mweresa CK (2016). Enhancing attraction of African malaria vectors to a synthetic odor blend. J. Chem. Ecol..

[22] Lalouette L (2016). Unexpected effects of sublethal doses of insecticide on the peripheral olfactory response and sexual behavior in a pest insect. Environ. Sci. and Pollut. Res. Int..

[23] Rinaldi F, Hanieh PN, Longhi C, Carradori S (2017). Neem oil nanoemulsions: characterisation and antioxidant activity. J. Enzyme Inhib. Med. Chem..

[24] Yarali A (2009). Odour intensity learning in fruit flies. Proc. Biol. Sci..

[25] Mishra D (2013). Olfactory memories are intensity specific in larval. Drosophila. J. Exp. Biol..

[26] Gerber B, Stocker RF, Tanimura T, Thum AS (2009). Smelling, tasting, learning: *Drosophila* as a study case. Results Probl. Cell Differ..

[27] Brown EB, Patterson C, Pancoast R, Rollmann SM (2017). Artificial selection for odor-guided behavior in *Drosophila* reveals changes in food consumption. BMC Genomics..

[28] Durisko Z, Anderson B, Dukas R (2014). Adult fruit fly attraction to larvae biases experience and mediates social learning. J. Exp. Biol..

[29] Hoffmann AA, Parsons PA (1984). Olfactory response and resource utilization in *Drosophila*: interspecific comparisons. Biol. J. Linnean Soc..

[30] Krishnan B, Dryer SE, Hardin PE (1999). Circadian rhythms in olfactory responses of *Drosophila melanogaster*. Nature..

[31] Zhou X, Yuan C, Guo A (2005). *Drosophila* Olfactory Response Rhythms Require Clock Genes but Not Pigment Dispersing Factor or Lateral Neurons. J. Biol. Rhythms..

[32] Revadi S (2015). Olfactory responses of *Drosophila suzukii* to host plant volatiles. Physiol. Entomol..

[33] Versace E (2016). Physiological and behavioral responses in *Drosophila melanogaster* to odorants present at different plant maturation stages. Physiol. Behav..

[34] Renn SC, Park JH, Rosbash M, Hall JC, Taghert PH (1999). A pdf neuropeptide gene mutation and ablation of PDF neurons each cause severe abnormalities of behavioral circadian rhythms in *Drosophila*. Cell..

[35] Deepak SN, Vedha Hari BN (2013). Optimization, Development and evaluation of Microemulsion for the release of combination of Guaifenesin and Phenylephrine. J. App. Pharm. Sci..

[36] Gaudry Q, Nagel KI, Wilson RI (2012). Smelling on the fly: sensory cues and strategies for olfactory navigation in *Drosophila*. Curr. Opin. Neurobiol..

[37] Devaud JM, Acebes A, Ferrús A (2001). Odor Exposure Causes Central Adaptation and Morphological Changes in Selected Olfactory Glomeruli in *Drosophila*. J. Neurosci..

[38] Benzer S (1967). Behavioral Mutants of *Drosophila* Isolated by Counter current Distribution. PNAS..

