# Supplementary material for: Evidence of nanoemulsion as an effective control measure for fruit flies Drosophila melanogaster
Source: Sci Rep. 2019 Jul 22;9:10578. doi: 10.1038/s41598-019-47045-3 (PMC6646352; doi:10.1038/s41598-019-47045-3)
Supplement: Supplementary file 1 — Supplementary figures [file 41598_2019_47045_MOESM1_ESM.pdf]

Evidence of nanoemulsion as an effective control measure for fruit flies *Drosophila melanogaster*

Sudhakar Krittika[1!], Indhumathi P[2!], Vedha Hari BN[2*], Ramya Devi D[2], Pankaj Yadav[1*]

1. Fly Laboratory # 210, Anusandhan Kendra-II, School of Chemical & Biotechnology, SASTRA Deemed to be University, Thanjavur-613401, Tamil Nadu, India.

2. Pharmaceutical Technology Laboratory # 214, Anusandhan Kendra-II, School of Chemical & Biotechnology, SASTRA Deemed to be University, Thanjavur-613401, Tamil Nadu, India.

!-These authors contributed equally.

*- Authors for correspondence.

E-mails:

Sudhakar Krittika-krittikacbe@gmail.com

Indumathi P- indhumaha97@gmail.com

Vedha Hari BN - vedhahari@scbt.sastra.edu

Ramyadevi D - ramya@scbt.sastra.edu

Pankaj Yadav-pankajyadav@scbt.sastra.edu

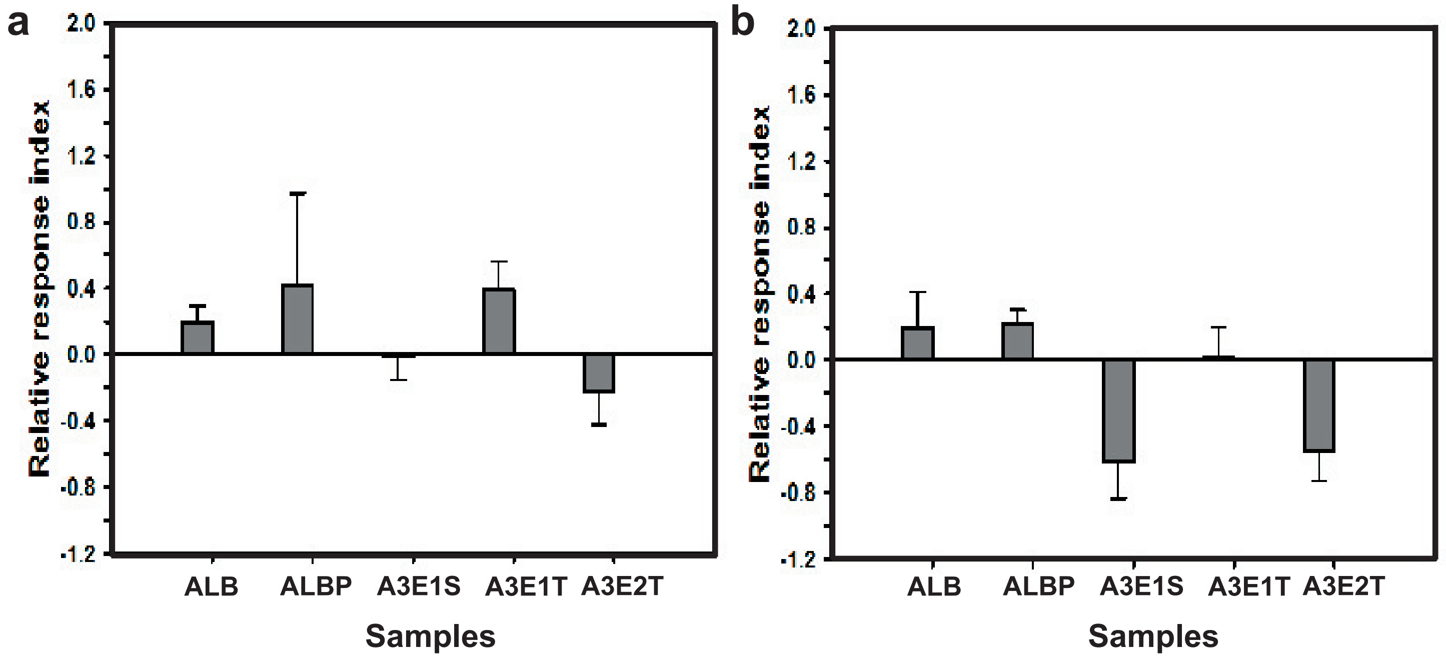

Figure Legend

Fig. 1. The chemotactic behaviour of flies upon exposure to different test samples in the morning (a) and evening (b) irrespective of the sex difference are indicated as their Relative Response Index (RRI). The Kruskal-Wallis test revealed no significant effect of sample in the morning $(H_{4,30}) = 3.742630$, $p = 0.4420$) but significant effect in the evening $(H_{4,30}) = 12.43303$, $p = 0.0144$).

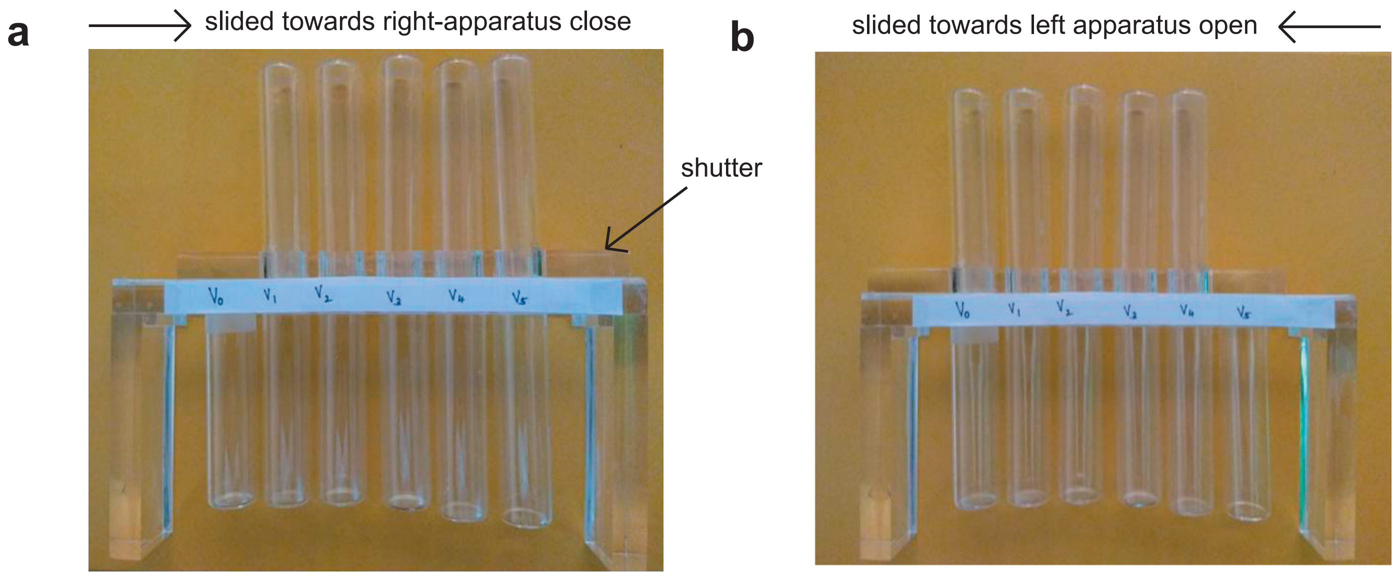

Figure Legend

Fig. 2. The counter current apparatus used for the behavioral studies of *D. melanogaster* upon exposure to tested samples. The apparatus is closed when the shutter is slided right (a), while it opens when it is slided left (b).
